# Graphene Fluoride: A Stable Stoichiometric Graphene Derivative and its Chemical Conversion to Graphene

**DOI:** 10.1002/smll.201001401

**Published:** 2010-11-22

**Authors:** Radek Zbořil, František Karlický, Athanasios B Bourlinos, Theodore A Steriotis, Athanasios K Stubos, Vasilios Georgakilas, Klára Šafářová, Dalibor Jančík, Christos Trapalis, Michal Otyepka

**Affiliations:** Regional Center of Advanced Technologies and Materials, Department of Physical Chemistry, Faculty of Science, Palacky University, tr. 17. listopadu 12, Olomouc 77146Czech Republic E-mail: michal.otyepka@upol.cz; Institute of Materials Science, NCSR “Demokritos”, Ag. Paraskevi AttikisAthens 15310, Greece E-mail: bourlinos@ims.demokritos.gr; Institute of Physical Chemistry, NCSR “Demokritos”, Ag. Paraskevi AttikisAthens 15310, Greece; Institute of Nuclear Technology and Radiation Protection, Environmental Research Laboratory, NCSR “Demokritos” Ag. Paraskevi AttikisAthens 15310, Greece

**Keywords:** Graphene, graphane, DFT, bandgap, fluorographene

## Abstract

Stoichoimetric graphene fluoride monolayers are obtained in a single step by the liquid-phase exfoliation of graphite fluoride with sulfolane. Comparative quantum-mechanical calculations reveal that graphene fluoride is the most thermodynamically stable of five studied hypothetical graphene derivatives; graphane, graphene fluoride, bromide, chloride, and iodide. The graphene fluoride is transformed into graphene via graphene iodide, a spontaneously decomposing intermediate. The calculated bandgaps of graphene halides vary from zero for graphene bromide to 3.1 eV for graphene fluoride. It is possible to design the electronic properties of such two-dimensional crystals.

## 1. Introduction

Two-dimensional atomic crystals such as graphene, NbSe_2_, Bi_2_Sr_2_CaCu_2_O_x_, MoS_2_, and WS_2_ represent recent advances in materials science, and have aroused growing interest from both academic and applied researchers.^1^ Apart from their fine structure, impressively low thickness, and delicate morphology at the atomic scale, these unique materials may also afford avenues to obtain important new information on low-dimensional systems based on quantum-size effects and, in turn, novel technological implementations.^1^ Conceptually, free-standing atomic layers of any kind can be directly separated from a corresponding multilayered material consisting of stacks of several single sheets by mechanical, physical, or chemical means in a nondestructive manner.^1^

In addition, graphite fluoride is a well-known covalent derivative of graphite with interesting electrochemical and electronic properties and potential applications in hydrogen storage.^2^ Depending on the preparative conditions employed, graphite fluoride with different fluorine-to-carbon atomic ratios can be obtained, the most representative example being the stoichiometric carbon monofluoride. This gray solid lacks the aromatic character of graphite, instead adopting a puckered cyclohexane-ring structure, with each carbon bearing a fluorine alternately above and then below the ring.^3^ Analogously to graphite oxide, which serves as a source of graphene oxide monolayers,^4^ graphite fluoride can be used as a precursor for another, less intensively studied graphene derivative: fluorinated graphene. While graphite fluoride has been examined as a starting material for making graphenes,^5^ studies on the isolation and theoretical aspects of individual, free-standing graphene fluoride (CF) monolayers have not yet been reported. Cheng et al. recently reported a synthesis of multilayered graphene fluoride,^6^ but evidence of the synthesis of monolayers is still lacking. Similarly, nothing is known about the synthesis and stability of other graphene halides (CX); indeed, we do not even know whether they can exist. Graphene fluoride is one of the thinnest materials in its class; nevertheless, it is expected to retain the interesting electrochemical and electronic properties of the bulk solid. It may therefore be useful in new technological applications pertinent to batteries or wide-bandgap semiconductors.2a,b Liquid-phase exfoliation of layered solids has emerged as a versatile and nondestructive technique for the isolation of dispersible single sheets of high quality from layered materials, and has been successfully employed as a straightforward method for the production of graphenes from graphite.^7^ It seems reasonable to suppose that this approach could be extended to other layered materials in which there are noncovalent interactions between adjacent layers, including graphite fluoride. The proposed liquid-phase exfoliation approach has numerous advantages: it is relatively simple, the monolayers obtained could be solvent-processed into various forms of interest in engineering (colloids, thin films, and composites), and proper handling of the dispersion should afford a material with a relatively high monolayer content.^7^

Herein, we demonstrate that the liquid-phase exfoliation of fluorinated graphite using sulfolane as the solvent affords single sheets of colloidal graphene fluoride, as evidenced by transmission electron microscopy (TEM), high-resolution transmission electron microscopy (HRTEM), selected area electron diffraction (SAED), and atomic force microscopy (AFM). Comparative theoretical calculations on a series of hypothetical graphene derivatives (graphane and graphene fluoride, chloride, bromide, or iodide) suggest that the thermodynamic stability of the fluorinated layers is significantly greater than that of the other derivatives, making their neat isolation from the pristine layered solid by solvent extraction a realistic goal. Theoretical calculations also demonstrate that electronic properties of graphene derivatives, such as the bandgap, can be tuned by varying their substituents. The differences in thermodynamic stability between the graphene halides can be exploited: CF can be converted via halide exchange to graphene iodide (CI), which is unstable and undergoes spontaneous decomposition to afford carbon nanostructures (graphene and nanodiamond).

## 2. Experimental and Computational Details

### 2.1. Production of CF Monolayers

In a typical procedure, 250 mg of purchased graphite fluoride (C_1_F_1_, Aldrich) was suspended in 50 mL sulfolane (Alfa Aesar, 99%) and the mixture was sonicated in a 135 W sonicator bath for 1 h at 50 °C. The resulting suspension was left to rest for two weeks at 80 °C in order to allow any insoluble particles to settle out. Then, the dilute, colloidally dispersed supernatant was carefully collected for microscopic analysis. Note that sulfolane is normally a solid under ambient conditions (mp: 28 °C). Therefore, in order to maintain its fluidity throughout our experimental process, it was necessary to keep the working temperature >40 °C.

### 2.2. Transformation of CF Monolayers into Graphene

KI (2 g) was completely dissolved in 50 mL of colloidal-sulfolane dispersion and the solution was heated under stirring at 240 °C for 2 h. The resulting precipitate was centrifuged and washed several times with water (4 × 50 mL) and ethanol (2 × 50 mL), each wash being followed by further centrifugation.

### 2.3. Characterization

TEM and HRTEM were carried out using JEOL JEM2010 and 3010 microscopes operating at 200 and 300 kV, respectively. The instruments were each equipped with a LaB6 cathode, an energy dispersive X-ray (EDX) detector. A very dilute drop of the colloidal dispersion was placed on a carbon-coated copper grid and allowed to dry by evaporation at ambient temperature. AFM images were obtained using an NTEGRA Aura (NT-MDT) microscope. A sample of the diluted dispersion was placed on synthetic mica as an atomically smooth support and evaporated at room temperature. The measurements were performed in air at room temperature in noncontact mode, with Si tips of the 1650–00 type at resonance frequencies ranging from 180 to 240 kHz. X-ray powder diffraction (XRD) experiments were performed with a PANalytical X´Pert PRO instrument (CoK*α* radiation) equipped with an X´Celerator detector. Samples were spread on zero-background Si slides and scanned in the 2*θ* range of 10–100° in steps of 0.017° for 720 s per step. IR spectra were acquired using a Fourier-transform infrared (FTIR) spectrometer (Bruker Equinox 55/S) and KBr pellets. Raman spectra were obtained using an inVia Reflex microRaman spectrometer (Renishaw) with a 100× objective lens and a crystal laser excitation of 514.5 nm operating at 0.1 mW.

### 2.4. Modeling

**Figure 1:**
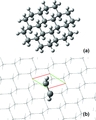
Models of chair like graphane and its halogen-substituted analogues: a) the finite model C_24_X_36_ for X = H, F, Cl, Br; b) the infinite (PBC) model; the 2D unit cell (C_2_X_2_) is shown and the translation vectors are indicated.

Theoretical calculations were performed on two different computational models of the chair conformation of graphane (CH) and on related halogenated systems (CX, X = F, Cl, Br, I). The first model (henceforth denoted model A) consists of perhydrocoronene and its halogenated analogues, i.e., C_24_X_36_ where X = H, F, Cl, Br (see Figure 1a). The second model (model B) consists of an infinite 2D structure with the smallest possible unit cell, *trans*-C_2_X_2_, under periodic boundary conditions (PBC) (see Figure 1b). All calculations were performed using gaussian 09^8^ in gas phase. Infinite structures were modeled using linear-scaling density-functional theory with Gaussian orbitals and PBC,^9^ rather than the plane-wave basis-set approach that is commonly applied in solid-state calculations. The Becke–Lee–Yang–Parr (BLYP) functional^10^ was employed in combination with the LANL2DZ^11^ basis set and core potential in most of the calculations discussed herein. All-electron calculations with the small 3–21G basis set were also performed, for comparative purposes. Structures corresponding to energy minima were obtained by optimization of all coordinates (and unit cell lengths in model B) using the default convergence criteria in Gaussian. A *k*-point mesh of 10 points × 10 points was used to sample the Brillouin zone at distances of 20 a.u. in each direction when setting up image cells; this was sufficient to obtain a convergence precision of 10^−6^ a.u. for total energies and 10^−4^ eV for bandgaps for CH, CF, and CCl. For CBr it was necessary to use a 40 point × 40 point *k*-point sampling to achieve the same convergence precision for total energies and a precision of 10^−3^ eV for bandgaps.

## 3. Results and Discussion

**Figure 2:**
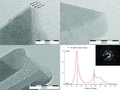
TEM images (top and bottom left) of CF sheets obtained after graphite fluoride exfoliation in sulfolane. Arrows in the HRTEM image (top left) indicate highly transparent graphene fluoride monolayers. The SAED pattern (bottom right) confirms the stoichiometry and structure of the layers corresponding to the original graphite fluoride.

Because of the unusually high polarity of sulfolane, which exceeds that of dimethylformamide (DMF) or dimethyl sulfoxide (DMSO), it is capable of dissolving a wide range of compounds, including hydrocarbons. TEM images reveal that the sheets obtained by single-step exfoliation of graphite fluoride with sulfolane are extraordinarily transparent, with lateral dimensions ranging between 200 nm and 2 μm (see Figure 2). Some of these sheets exhibit a twisted morphology, as is commonly observed in graphene monolayers.^7^ SAED analysis confirms that the crystal structure and stoichiometry of the original graphite fluoride are retained in the exfoliated sheets. On the other hand, the nature and transparency of these sheets is completely different from that of the pristine graphite fluoride, in which dark, multilayered, 3D sheets were observed (see Figure S1, Supporting Information). In this context, it is important to note that the structure of sulfolane-treated CF sheets (shown in the HRTEM image in Figure 2) consists of overlapping single layers of graphene fluoride rather than multilayered graphite fluoride, due to widely varying distances between the sheets.

**Figure 3:**
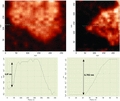
Two independent AFM images of graphene fluoride monolayers (top) and their height profiles (bottom), providing evidencing for the layers being <0.9 nm thick.

AFM data provide definitive proof of the presence of graphene fluoride monolayers, the thickness of which was generally <0.9 nm (see Figure 3). The monolayers comprise a significant fraction of the exfoliated graphene fluoride, although multilayered sheets ca. 2–4 nm thick are also detected. It is worth noting that determination of the sheet thickness from these AFM height profiles will be slightly affected by the signal noise and the presence of impurities (solvent) on the sheet surface and/or the mica support. The true values are likely to be lower than those observed, and are certainly less than the thickness of the two-layered graphene fluoride system, which we predict to be 1.24 nm, determined as the sum of the distance between the F–F planes in CF (0.33 nm, determined in the calculations discussed below), the van der Waals radius of fluorine (0.147 nm), and the interlayer distance in (CF)_2_ (0.62 nm as determined by Han et al.^12^). Using the same data, we estimate that the thickness of one CF layer amounts to 0.62 nm, which agrees well with our experimental observations (0.67–0.87 nm).

Ab initio theoretical calculations were used to elucidate the thermodynamical stability and structural features of graphene derivatives. The C–C bond lengths predicted on the basis of the two chosen models are similar for both CH and for CF, but they are ≈0.03 Å greater than those reported in the literature (see Supporting Information, Supplementary Tables S1 for CH^13^ and S2 for CF2a,^12^,^14^). The models are not in perfect agreement for the heavier substituents (Cl and Br); model B, which assumes an infinite layer, should be the more accurate of the two. Finally, CI seems to be highly unstable: optimization of this species using model A invariably resulted in the dissociation of the iodine atoms from carbon to form molecular iodine. The thermodynamic stability of CF, CCl, and CBr with respect to CH is indicated by the (free) energy change associated with the conversion of the hydrocarbon to the halogenated derivative. For model A the energy difference Δ*E* (Δ*G*) is calculated from the total energies of the reactants and products according to Equation 1:

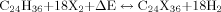
(1)
For model B, this energy change is calculated in terms of a single cell, i.e.,

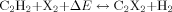
(2)

If the CX compound is more stable than CH, Δ*E* takes a negative value. Table 1 shows that CF is more stable than CH; this contradicts an earlier prediction that CH is at least as stable as CF.13a On the other hand, CCl and CBr are less stable than CH, but are not necessarily thermodynamically unstable compounds.

**Table 1:** Calculated values of graphane and graphene halides. The upper part of the table contains data pertaining to model A (perhydrocoronene and its perhalogenated analogues). Columns 2–4 contain distances in Å for one side of the CX structure; the geometrical parameters tabulated are those of the inner carbon ring. The X–X bond distance *d*′ in the appropriate optimized halogen dimer in the gas phase is shown for comparative purposes. The lower part of the table contains data pertaining to model B (the infinite structure). The crystal structure of the solid (model B) is described in terms of the X–C, C–C, and X–X distances (as per model A), and the length of the translation vectors (*TV*) where *a* = *b*; the angle between the vectors is 120°. The thermodynamic stabilities of CF, CCl, and CBr with respect to CH in terms of their energies of formation, Δ*E* (Δ*G*, 278 K) as defined in Equation 1 and 2 in kJ mol^−1^, are shown here, as are the direct bandgaps (*E*_g_) at the Γ point and the maximal direct gaps (*E*_gmax_) (in eV).

model A							

CX/Basis Set	*d*(X–C)	*d*(C–C)	*d*(X–X)	*d′*(X–X)	Δ*E*	Δ*G*	
CH/LANL2DZ	1.115	1.565	2.594	0.748	0	0	
CH/3–21G	1.110	1.562	2.586	0.751	0	0	
CF/LANL2DZ	1.452	1.596	2.715	1.492	−4653	−4795	
CF/3–21G	1.410	1.571	2.568	1.475	−5470	−5617	
CCl/LANL2DZ	1.899	1.702	3.087	2.254	2493	2309	
CCl/3–21G	1.895	1.679	3.061	2.261	2122	1932	
CBr/3–21G	1.994	1.759	3.266	2.503	4041	3813	

model B							

CX/Basis Set	*d*(X–C)	*d*(C–C)	*d*(X–X)	*TV*	Δ*E*	*E*_g_	*E*_gmax_
CH/LANL2DZ	1.115	1.562	2.579	2.58	0	5.50	12.64
CH/3–21G	1.110	1.560	2.568	2.57	0	5.37	12.90
CF/LANL2DZ	1.445	1.594	2.639	2.64	−228	3.08	9.96
CCl/LANL2DZ	1.867	1.794	2.985	2.99	332	0.95	6.79
CCl/3–21G	1.871	1.778	2.954	2.96	302	0.75	6.79
CBr/LANL2DZ	2.028	1.937	3.244	3.24	547	0.001a)	5.72
CBr/3–21G	1.991	1.918	3.209	3.21	497	0.002b)	6.05

a) Indirect gap −0.16 eV.

b) indirect gap −0.19 eV.

**Figure 4:**
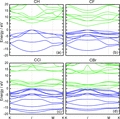
The electronic band structure in the vicinity of the bandgaps of CH (a), CF (b), CCl (c), and CBr (d) along lines connecting the high-symmetry points K, Γ, and M in the Brillouin zone. The zero-energy level has been set to the valence band maximum.

Table 1 summarizes the calculated electronic properties of the studied materials, i.e., their bandgaps (*E*_g_) and maximal direct gaps (*E*_gmax_). CH, CF, and CCl are direct bandgap materials, while CBr is an indirect bandgap material. The bottom of the conduction band and the top of the valence band are located at the Γ point in the first Brillouin zone for CH, CF, and CCl (see Figure 4); in these species, the top of the valence band is doubly degenerate. CH and CF have similar band structures, although CF has more valence bands. On the other hand, the structures of the top valence bands are almost the same for CF, CCl, and CBr, and the energies of the valence bands become closer as the bandgaps decrease. The maximum bandgaps of all of the species considered are located at K points. Graphane (CH) is a wide-gap semiconductor13a,c (or alternatively, a wide-gap insulator13d,e); using a very accurate GW method,^15^ Lebegue et al.13c estimated its bandgap to be 5.4 eV. The bandgap of CH determined in this work (5.5 eV, see Table 1) agrees with this value, however, such agreement might be coincidental. Nonetheless, the presented bandgap calculations are still meaningful, because the trends in bandgaps of different CX derivatives are expected to be correct. The bandgap width is considerably modulated by substitution and decreases as the atomic number of the halogen substituent increases. The calculated bandgap of CF is 3.1 eV (see Supporting Information for a comparison with recently performed plane-wave calculations14b), indicating that CF can be considered a wide-gap semiconductor. The chlorinated derivative (CCl) gave an even smaller bandgap of about 0.9 eV. Finally, the brominated derivative (CBr) has almost no bandgap (0.002 eV) and was found to have a negative indirect gap. Overall, these data indicate that by careful choice of substituents and modulation of the substituent lattice,^16^ it may be possible to fine-tune the electronic properties and thermodynamic stability of graphene derivates.

**Figure 5:**
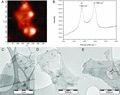
AFM image (A), Raman spectrum (B), and TEM images (C,D,E) of graphene sheets obtained by graphene fluoride conversion via a metastable CI intermediate. The thickness of the sheet in the AFM image is 0.6 nm. The ring in the TEM image indicates the area of the graphene surface covered by nanodiamond particles.

As the direct thermal transformation is quite difficult, the differences in the stability of the graphene halides alone would be beneficial for a targeted two-step conversion of graphene fluoride to graphene due to the relatively high stability of CF layers. This is supported by the fact that neither thermal heating of the neat sulfolane dispersion with exfoliated CF layers at 240 °C, nor their solid-state heating at the same temperature, result in any changes in the XRD and IR spectra. However, if potassium iodide is added to the colloidal CF dispersion in sulfolane and the mixture thus obtained is heated at 240 °C, extensive precipitation is observed. The Raman spectrum (see Figure 5) and XRD pattern (see Supporting Information, Figure S2) of this precipitate are consistent with those of graphite/graphene. More specifically, the Raman spectrum of the product shows the typical D (1350 cm^−1^) and G (1585 cm^−1^) bands of carbon materials. This spectrum is dramatically different from that observed for the pristine fluorinated graphite.^17^ In addition, taking into account the broadness and relative intensities of the peaks, the spectrum is quite similar to the Raman profiles of several other reduced graphite oxide derivatives.^18^ Very transparent sheets, indicative of the presence of graphenes, were detected by TEM; similar sheets with a thickness ranging between 0.5 and 0.68 nm were observed by AFM (see Figure 5). Intriguingly, the surface of these sheets is covered with ultrasmall nanoparticles, which were identified as nanodiamonds by SAED analysis (see Supporting Information, Figure S2). The proportion of these nanodiamond particles in the material as a whole seems to be relatively small, as they were not observed by XRD. The structure of graphite fluoride suggests a possible explanation for the formation of nanodiamonds as a side-product. Solid CF exhibits a diamondlike covalent network of sp^3^ carbons. Hence, elimination of the halide substituents and the subsequent condensation of adjacent layers might provide a pathway for the growth of a diamondlike phase. Clearly, the ease with which this elimination can occur will be dependent on the halide, e.g., iodine might be expected to be more labile than fluorine. Thus, the graphene fluoride sheets can be simply transformed to metastable graphene iodide, which quickly disproportionates to carbon and iodine (see Equation 3,4):

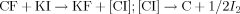
(3)

To confirm this reaction mechanism, we reacted CF with KI using DMF as the solvent; under these conditions, the reaction occurs at just 150 °C. The IR spectrum of the reaction mixture was monitored; over the course of the reaction, the peak corresponding to the C–F stretch at 1220 cm^−1^ disappears and a new peak corresponding to the C–I unit (745 cm^−1^) is observed (see Figure S3, Supporting Information). It seems that at this lower reaction temperature, the CI intermediate is relatively long-lived and the thermal elimination of iodine is comparatively slow.

## 4. Conclusion

This study provides the first experimental and theoretical evidences for the existence of thermodynamically stable, stoichiometric graphene derivative: graphene fluoride. The bandgap width of this monolayered graphene derivative have been compared with those calculated for graphane and other graphene halides. The halide-exchange process can be exploited for the transformation of graphene fluoride to graphene, with nanodiamonds being formed as a minor by-product.

*Note added at proof:* During the review process other papers dealing with various “graphene fluoride” systems were published.^19^ However, the fully stoichiometric graphene fluoride was prepared by mechanical exfoliation^20^ and liquid-phase exfoliation (this paper), at the same time.
