# Supplementary material for: Graphene Fluoride: A Stable Stoichiometric Graphene Derivative and its Chemical Conversion to Graphene
Source: Small. 2010 Nov 22;6(24):2885–91. doi: 10.1002/smll.201001401 (PMC3020323; doi:10.1002/smll.201001401)
Supplement: Supplementary file 1 [file smll0006-2885-SD1.pdf]

Copyright WILEY-VCH Verlag GmbH & Co. KGaA, 69469 Weinheim, Germany, 2010.

Supporting Information

for *Small*, DOI: 10.1002/smll. 201001401

Graphene Fluoride: A Stable Stoichiometric Graphene Derivative and its Chemical Conversion to Graphene

Radek Zborˇil, Frantisˇek Karlickyˊ, Athanasios B. Bourlinos,* Theodore A. Steriotis, Athanasios K. Stubos, Vasilios Georgakilas, Klára Sˇafárˇová, Dalibor Jancˇík, Christos Trapalis, and Michal Otyepka*

DOI: 10.1002/smll.201001401

Supplementary Information to Graphene fluoride: a stable stoichiometric graphene derivative and its chemical conversion to graphene

by

R. Zbořil, F. Karlický, A. B. Bourlinos, T. A. Steriotis, A. K. Stubos, V. Georgakilas, K. Šafářová, D. Jančík, C. Trapalis, M. Otyepka

Supplementary Table S1 Plane-wave basis set calculations on chair conformation of graphane (CH) available from literature. Distances are given in Å, band gaps in eV. Abbreviations used: PP – pseudopotential, PAW – projector augmented waves, TV – length of translation vector. For references see the main text.

	Sofo et al.	Lebegue et al.	Liu and Shen	Lu and Feng [a]	Flores et al.
code	CASTEP	VASP	ABINIT	DMOL3	DMOL
method	DFT (GGA)	GWA	DFT (LDA)	DFT (GGA)	DFT (GGA)
core-electrons	PP	PAW	PAW	all-electron	all-electron
d(C-C)	1.52	1.53	---	1.54	1.537
d(C-H)	1.11	1.11	---	1.11	---
TV	2.516	---	---	2.463	2.540
band gap	3.5	5.4	3.6	~ 4.4	---

[a]Calculation on graphene nanoribbons.

Supplementary Table S2 Plane-wave basis set calculations on chair conformation of graphene fluoride (CF) available from literature. For details see caption of Supplementary Table S1. For references see the main text.

	Charlier et al. [a]	Tagaki and Kusakabe [a]	Han et al. [a]	Zhou et al.
code	CORNING	FHI	SEQQUEST	VASP
method	DFT (LDA)	DFT (LDA)	DFT (GGA)	DFT (LDA)
core-electrons	PP	PP	PP	PAW
d(C-C)	1.552	---	---	1.553
d(C-F)	1.37	---	1.38	1.365
TV	2.553	2.55	2.61	---
band gap	3.5	~ 3.0	---	2.96

[a] Calculation on graphite fluoride.

Supplementary Figure S1 TEM image of the pristine graphite fluoride and corresponding selected area electron diffraction.

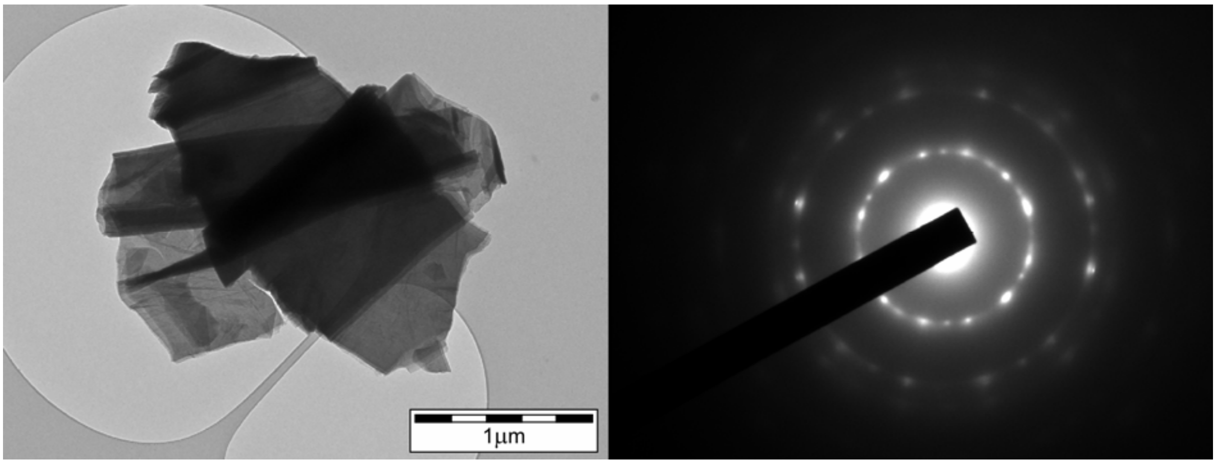

Supplementary Figure S2 XRD (left) and SAED pattern (right) of the carbon nanostructures obtained after halide exchange process and spontaneous decomposition of graphene iodide.

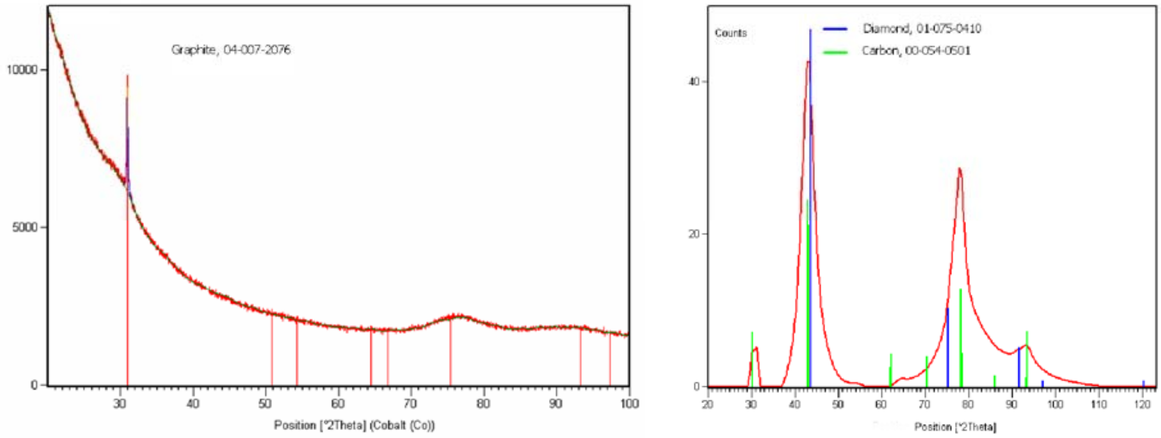

Supplementary Figure S3 IR spectra demonstrating the considerably faster kinetics of the CF→ CI→ C transformations in sulfolane (left) compared to DMF (right).

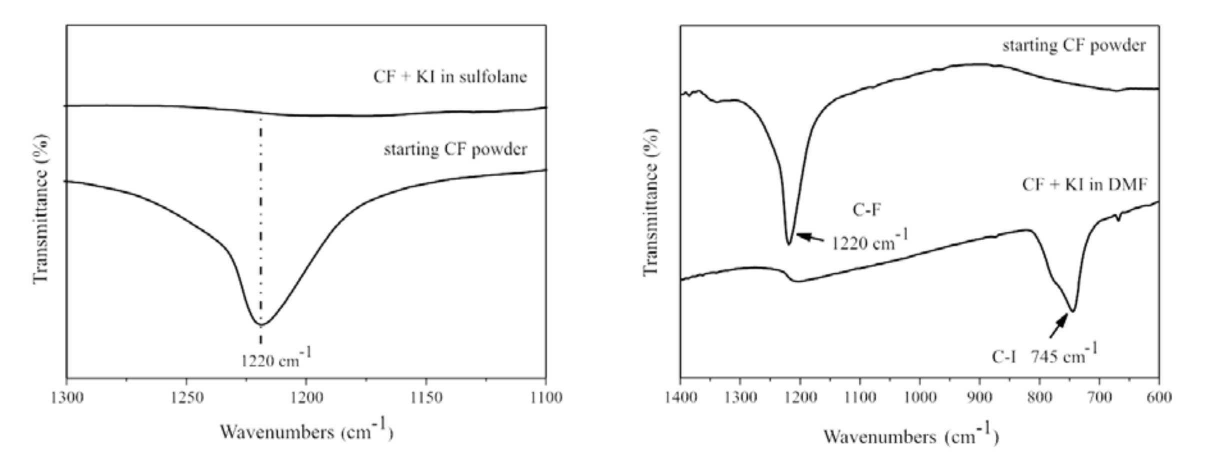

Full reference 8:

Frisch, M. J., Trucks, G. W., Schlegel, H. B., Scuseria, G. E., Robb, M. A., Cheeseman, J. R., Scalmani, G., Barone, V., Mennucci, B., Petersson, G. A., Nakatsuji, H., Caricato, M., Li, X., Hratchian, H. P., Izmaylov, A. F., Bloino, J., Zheng, G., Sonnenberg, J. L., Hada, M., Ehara, M., Toyota, K., Fukuda, R., Hasegawa, J., Ishida, M., Nakajima, T., Honda, Y., Kitao, O., Nakai, H., Vreven, T., Montgomery, Jr., J. A., Peralta, J. E., Ogliaro, F., Bearpark, M., Heyd, J. J., Brothers, E., Kudin, K. N., Staroverov, V. N., Kobayashi, R., Normand, J., Raghavachari, K., Rendell, A., Burant, J. C. Iyengar, S. S. Tomasi, J. Cossi, M. Rega, Millam, N. J., Klene, M. Knox, J. E., Cross, J. B., Bakken, V., Adamo, C., Jaramillo, J., Gomperts, R. E. Stratmann, O. Yazyev, A. J. Austin, R. Cammi, C. Pomelli, J. W. Ochterski, R. Martin, R. L., Morokuma, K., Zakrzewski, V. G., Voth, G. A., Salvador, P., Dannenberg, J. J., Dapprich, S., Daniels, A. D., Farkas, O., Foresman, J. B., Ortiz, J. V., Cioslowski, J., and Fox, D. J. Gaussian 09, Revision A.02, Gaussian, Inc.: Wallingford CT, 2009.
